# Characterization of Skeletal Muscle Endocrine Control in an In Vitro Model of Myogenesis

**DOI:** 10.1007/s00223-020-00678-3

**Published:** 2020-02-27

**Authors:** Cecilia Romagnoli, Roberto Zonefrati, Preeti Sharma, Marco Innocenti, Luisella Cianferotti, Maria Luisa Brandi

**Affiliations:** 1 Department of Experimental and Clinical Biomedical Sciences, University of Florence, Largo Palagi 1, 50139 Florence, Italy; 2 Department of Health Sciences, University of Florence, Viale Pieraccini 6, 50139 Florence, Italy

**Keywords:** Skeletal muscle, Satellite cells, Myogenesis, Skeletal muscle endocrinology, Hormone receptors

## Abstract

Skeletal muscle has remarkable regenerative abilities regulated by a highly orchestrated process involving the activation of cellular and molecular responses, which are dependent on satellite cells. These cells maintain the stem cell population and provide numerous myogenic cells that proliferate, differentiate, fuse and lead to new myofiber formation for a functional contractile tissue. We have isolated and characterized satellite cells obtained from human biopsies and established an in vitro model of myogenesis, evaluating muscle regeneration, monitoring the dynamic increases of the specific myogenic regulatory factors and the final formation of multinucleated myofibers. As the skeletal muscle is an endocrine tissue able of producing many substances that can act on distant organs, and it can be physiologically modulated by a variety of hormones, we embarked in a project of characterization of muscle cell endocrinology machinery. The expression of a large array of hormone receptors was quantified during the process of myogenesis. The results obtained showed a significant and generalized increase of all the tested hormone receptors along the process of differentiation of human cultured cells from myoblasts to myocytes. Interestingly, also the production of the myokine irisin increased in a parallel manner. These findings point to the human cultured myoblasts as an ideal model to characterize the skeletal muscle endocrine machinery and its hormonal regulation.

## Introduction

Skeletal muscle is the most abundant tissue in the human body, accounting for about 40–45% of total body weight. It plays an important role in controlling physical activity, including voluntary locomotion, postural behavior, and breathing. Moreover, it has an extraordinary ability to adapt to physiological demands, such as growth, and to regenerate new muscle fibers after damage by injury or intense physical activity [1].

The regeneration and remodeling of skeletal muscles are extremely complex biological processes, in which skeletal muscle stem cells (also known as satellite cells, SCs) are involved. The SCs are located under the basal lamina of the myofiber; this position, between the myofiber and the surrounding extracellular matrix (ECM), is the reason Alexander Mauro gave them this name in 1961 [2]. In healthy adult mammalian muscle, SCs are predominantly quiescent (phase G_0_) and represent 2.5–6% of all nuclei of a given fiber; however, after injury or degeneration, SCs become activated and can generate large numbers of new myofibers within just few days [3]. Like stem cells, satellite cells also self-renew to maintain their own population, re-establishing their numbers and quiescent state by homing back to highly specialized niches, thus allowing future rounds of regeneration [4]. Specific temporal factors, called Myogenic Regulatory Factors (MRFs), members of the basic helix-loop-helix (bHLH) family of transcription factors including Myf-5, MyoD1, Myogenin and MRF4, are an essential group of four muscle-specific proteins responsible for acting at multiple points in the muscle lineage to cooperatively establish the skeletal muscle phenotype [4, 5].

Besides its well-known structural and biomechanical functions for the purpose of movement, the skeletal muscle is considered a secretory organ, capable of producing several substances called myokines, which can act on the muscle itself, on nearby tissues, and on distant organs, in an autocrine, paracrine and endocrine fashion, respectively [6–8]. These functions can be physiologically modulated by physical stimuli and by cytokines, mineral ions and hormones, or can be modified by sarcopenia, morphological modification of muscle fibers and/or endocrinopathies.

The evaluation of the skeletal muscle as a secretory organ is not fully understood, neither it is the endocrine control of muscle function and differentiation [9]. A future chapter in the endocrine discipline will certainly be musclecrinology. The aim of our study was to evaluate the endocrine machinery in an in vitro cellular model of myogenesis obtained from human skeletal muscle biopsies. The understanding of hormonal production and regulation in the skeletal muscle remodeling may contribute to the identification of new possible therapeutic targets in pathologies in which the myogenesis and/or the function of mature myocytes is affected.

## Materials and Methods

### Isolation of Human Skeletal Muscle-Derived Cells (hSkMCs)

Primary cultures were isolated from human skeletal muscle biopsies of 3 healthy adult volunteers undergoing plastic surgery, after signing an informed consent in accordance with a protocol approved by the Local Ethics Committee of AOU Careggi, Firenze (Italy), for human studies (Rif. N. 14.017), as well as the ethical standards stated in the Declaration of Helsinki (1964) and its later amendments or comparable ethical standards. The minced specimens were processed within 3 h from the operation and enzymatically digested for 3 h at 37 °C in Ham’s F12 Coon’s modification medium (Sigma-Aldrich) supplemented with 20% fetal bovine serum (FBS) and 3 mg/ml collagenase type I (C-0130, Sigma-Aldrich). The tissues were then mechanically dispersed by pipetting and passed through a sterile 100 μm stainless steel tissue sieve to remove any large debris. The undigested tissue trapped in the sieve was discarded, while the infranatant containing the hSkMC fraction was collected and the cells sedimented by centrifugation at 300×*g* for 5 min. The cells were then pre-plated into 100 mm Petri dishes for 1 h at 37 °C to remove fibroblasts which adhere to plastic more avidly than satellite cells. Afterwards, the resulting suspension was seeded in 100 mm tissue culture plates at 37 °C in humidified atmosphere with 5% CO_2_ using a skeletal muscle cell growth medium (GM) composed of Skeletal Muscle Cell Basal Medium (PromoCell GmbH, cod. C-23260) supplemented with 5% Fetal Calf Serum (FCS), 50 µg/ml fetuin, 10 ng/ml Epidermal Growth Factor (EGF), 1 ng/ml basic Fibroblast Growth Factor (bFGF), 10 µg/ml insulin, 0.4 µg/ml dexamethasone and 100 IU/ml penicillin, 100 μg/ml streptomycin (Table 1). The medium was refreshed twice a week and the cells were used for further subculturing or cryopreservation upon reaching 5 × 10^3^ cells/cm^2^. Cells at the early passages (from 1 to 4) were used for all the experiments. All tissue culture plates utilized for cell amplification and experiments were previously coated with Matrigel® (BD Biosciences) in order to increase cell attachment, proliferation and to maintain cell phenotype [10].

**Table 1:** Supplements and concentrations after their addition to the basal medium for growth medium and myogenic differentiation medium used in the experiments, respectively

	Growth medium	Myogenic differentiation medium
Fetal calf serum	0.05 ml/ml	–
Fetuin	50 µg/ml	–
Epidermal growth factor	10 ng/ml	–
Basic fibroblast growth factor	1 ng/ml	–
Insulin	10 µg/ml	10 µg/ml
Dexamethasone	0.4 µg/ml	–

### hSkMC Characterization and Multipotency Evaluation

The characterization of the hSkMC cell lines was performed analyzing the presence of the surface markers of mesenchymal stem cells and specific markers of the isolated satellite cells by flow cytometry analysis and by studying their multipotency toward the myogenic, the adipogenic and the osteogenic phenotypes, as previously described [11].

#### Flow Cytometry

hSkMC lines were evaluated by flow cytometry with a CyFlow®Space cytometer (Sysmex Partec), equipped with FlowMax® software. The antibodies used (Abcam) were directed against the following antigens (the tags are given in parentheses): CD44 (PE/Cy7), CD90 (APC), CD105 (FITC), CD45 (PerPC), CD34 (PE) and CD56 (PerCP Cy5.5) and PAX-7 (FITC). Each antibody was diluted according to manufacturer's instruction. Briefly, 1 × 10^5^ cells were labeled with antibodies in PBS with 1% bovine serum albumin (BSA) for 20 min RT in the dark, then washed once and promptly analyzed.

#### Myogenic Differentiation

hSkMC lines at 70% of confluence were cultured with a specific myogenic medium (MM): Skeletal Muscle Cell Basal Medium (PromoCell GmbH, cod. C 23260) supplemented with 10 µg/ml insulin, 100 IU/ml penicillin and 100 μg/ml streptomycin. The medium was refreshed twice a week. The expression of the myogenic phenotype was evaluated by microscopic observations of the multinucleated cells formation, by immunofluorescence of the myosin heavy chain (MHC) and by gene expression analysis, after 10 days of induction (Table 1).

#### Adipogenic Differentiation

hSkMC lines were cultured with a specific adipogenic medium (AM): Ham’s F12 Coon’s modification medium supplemented with 10% (FBS), 100 IU/ml penicillin, 100 μg/ml streptomycin and 1 μM dexamethasone, 1 μM bovine insulin, 0.5 mM isobutylmethylxanthine (IBMX). The medium was refreshed twice a week. The expression of the adipogenic phenotype was evaluated on cells cultured in AM for 10 days by cytochemical staining with Oil Red O and brightfield observations (Axiovert 200, Zeiss).

#### Osteogenic Differentiation

hSkMCs were plated on tissue culture dishes at a cell density of 1 × 10^4^ cells/cm^2^ and grown to 70–80% confluence. Afterwards, the medium was switched to osteogenic medium (OM): Ham’s F12 Coon’s modification medium supplemented with 10% FBS, 100 IU/ml penicillin, 100 μg/ml streptomycin, 10 nM dexamethasone, 0.2 mM sodium l-ascorbyl-2-phosphate, and 10 mM β-glycerol phosphate. The medium was refreshed twice a week. The expression of the osteoblastic phenotype was evaluated at 20 days from induction by monitoring the production of mineralized nodules by cytochemical staining. The cells were washed with DPBS (two times), fixed in 4% paraformaldehyde (PFA)/DPBS for 15 min, and washed with ultrapure water (three times). Calcium mineral deposits were stained with 1 µg/ml calcein added to the OM and nuclei were counterstained with 1 µg/ml bisbenzimide for 5 min; calcium mineralized deposits were stained in fluorescent green, nuclei in blue, and then visualized in epifluorescence microscopy (Axiovert 200, Zeiss).

### Immunofluorescence

hSkMCs were seeded into 24-well plates (1 × 10^4^ cells/well) and cultured for 24 h in GM. Afterwards, cells were fixed for 10 min with 4% paraformaldehyde and permeabilized for 10 min with 0.2% Triton X 100 at RT. Cells were treated for another 30 min at 37 °C with RNAse in 2% bovine serum albumin (BSA) in order to degrade RNA and block non-specific sites. Samples were then incubated overnight with primary antibody for PAX-7 and MHC (Abcam, Cambridge, UK) in PBS at 4 °C. After extensive washes with PBS, goat anti-mouse IgG (H + L) SuperClonal secondary antibody, Alexa Fluor 488 conjugate (Thermo Fischer Scientific, Waltham, MA, USA) was incubated for 1 h at room temperature in the dark. Subsequently, nuclei were counterstained with 10^−5^ M propidium iodide. Samples were then washed with PBS for observation in laser scanning confocal microscopy (LSCM), using an LSM 510 Meta microscope (ZEISS, Oberkochen, Germany) [12].

### RNA Extraction and Real-Time qPCR Analysis

Gene expression analysis in the hSkMCs was performed in GM and after 9 days of induction in MM. The genes included in the analysis were PAX-7, MyoD-1, Myf-5, MRF-4, Myogenin, Desmin, MHC, Irisin, and specific hormone receptor genes (VDR, TRα, TRβ, GCR, IGF-1, PTH1R, LRP-5, LRP-6). Target gene expression was normalized to 40S ribosomal protein S18 (RPS18). All procedures for amplification were previously described [13] (Table 2).

**Table 2:** Primers and TaqMan probes used for the experiments

Gene	Primer sequences (5′–3′) and TaqMan probes	Amplicon size (bp)	*T*_m_ (°C)
PAX-7 for	GGTACCGAGAATGATGCGG	124	55
PAX-7 rev	CCCATTGATGAAGACCCCTC		
Pax-7 Probe	6-FAM/AGCTGATTG /Zen/ACCCGGCCTTGG/3IABkFQ		
MyoD-1 for	GACGTGCCTTCTGAGTCG	148	55
MyoD-1 rev	CTCAGAGCACCTGGTATATCG		
MyoD-1 Probe	6-FAM/CGCTGCTCT/Zen/CTCCCTCGCTG/3IABkFQ		
Myf-5 for	ATGCCATCCGCTACATCG	145	55
Myf-5 rev	ACAGGACTGTTACATTCGGC		
Myf-5 Probe	6-FAM/CCCCACCTC/Zen/CAACTGCTCTGAT/3IABkFQ		
MRF-4 for	CCCTGGAATGATCGGAAACA	95	55
MRF-4 rev	CTTCAGCTACAGACCCAAACA		
MRF-4 Probe	6-FAM/ATCTTGAGG/ZEN/GTGCGGATTTCCTGC/3IABkFQ		
Myogenin for	AGCGAATGCAGCTCTCAC	150	55
Myogenin rev	TGTGATGCTGTCCACGATG		
Myogenin Probe	6-FAM/TGACCCTAC/Zen/AGATGCCCACAACC/3IABkFQ		
MHC for	GAGTCCTTTGTGAAAGCAACAG	143	55
MHC rev	GCCATGTCCTCGATCTTGTC		
MHC Probe	6-FAM/CAAGTCTTC/Zen/CCCATGAACCCTCCC/3IABkFQ		
Desmin for	AACGCGATCTCCTCGTTG	101	55
Desmin rev	GAGAACAATTTGGCTGCCTTC		
Desmin Probe	6-FAM/CAATTCTGC/ZEN/GCTCCAGGTCAATGC/3IABkFQ		
VDR for	CCGCATCACCAAGGACAA	112	62
VDR rev	CTTCCTCTGCACTTCCTCATC		
VDR Probe	6-FAM/TGTGGACAT/ZEN/CGGCATGATGAAGGA/3IABkFQ		
TRα for	TCCCTAGTTACCTGGACAAAGA	133	59
TRα rev	GGATGGAGGTTCTTCTGGATTG		
TRα Probe	6-FAM/ACAGCGGTA/ZEN/GTGATAACCAGTTGCC/3IABkFQ		
TRβ for	CTTCCAAACGGAGGAGAAGAA	115	59
TRβ rev	CGTGATACAGCGGTAGTGATAC		
TRβ Probe	6-FAM/TGTGTAGTG/ZEN/TGTGGTGACAAAGCCA/3IABkFQ		
GCR for	TGGTCCTGTTGTTGCTGTT	103	55
GCR rev	CTTCCCTGGTCGAACAGTTT		
GCR Probe	6-FAM/TAAGCTCTC/ZEN/CTCCATCCAGCTCCT/3IABkFQ		
IGF-1 for	CAGCAAGTGAGGAGAGGAAC	131	59
IGF-1 rev	GTGTGAGAAGACCACCATCAA		
IGF-1 Probe	6-FAM/TCGAAGAGA/ZEN/GCAAATGCACATCCCT/3IABkFQ		
LRP-5 for	CCCAGTCTGTCCAGTACATG	134	59
LRP-5 rev	CTCAGAGACCAACCGCATC		
LRP-5 Probe	6-FAM/CCAACCTCA/ZEN/ATG/3IABkFQ		
LRP-6 for	CCCATTTGTGTTTGATGTCTCC	137	60
LRP-6 rev	CAAGTCTGTCCTTCGAGCTAAA		
LRP-6 Probe	6-FAM/AAACCTGCA/ZEN/AAGATGGTGCCACAG/3IABkFQ		
PTH-1R for	GGGAAGCCCAGGAAAGATAAG	125	58
PTH-1R rev	CACAGGATGTGGTCCCATT		
PTH-1R Probe	6-FAM/TGCCTCCTT/ZEN/GTCCTCCTCAGACTC/3IABkFQ		
Irisin for	ACTATGTACTCCGTATCCTCCTC	126	55
Irisin Rev	TGTCATCGGATTTGCCATCT		
Irisin Probe	6-FAM/CCAGCAGAA/ZEN/GAAGGATGTGTCGGAT/3IABkFQ		
RPS18 for	GATGGCAAAGGCTATTTTCCG	132	60
RPS18 rev	TCTTCCACAGGAGGCCTAC		
RPS18 Probe	6-FAM/TTCAGGGAT(ZEN/CACTAGAGACATGGCTGC/3IABkFQ		

TaqMan probes with F as reporter fluorochrome (6-carboxyfluorescein [6-FAM]) and ZEN as quencher. Fluorochrome (Iowa Black FQ); *bp* base pairs of amplicon size; *T*_m_ melting temperature (°C)

### Statistical Analysis

All gene expression analyses were performed in tetraplicate and each experiment was repeated three times. All data were expressed as means ± SD and are the number of mRNA molecules of the specific genes normalized to the housekeeping RPS18 mRNA. Statistical differences among mean values were analyzed using ANOVA and a *post hoc* sequentially rejective multiple Bonferroni test, with predetermined (default) experimentwise probability α_T_ = 0.05, comparing two groups: the value of the specific gene after 9 days of myogenic induction with respect to the control in proliferating medium.

## Results

### Isolation and Characterization of hSkMCs

**Fig. 1:**
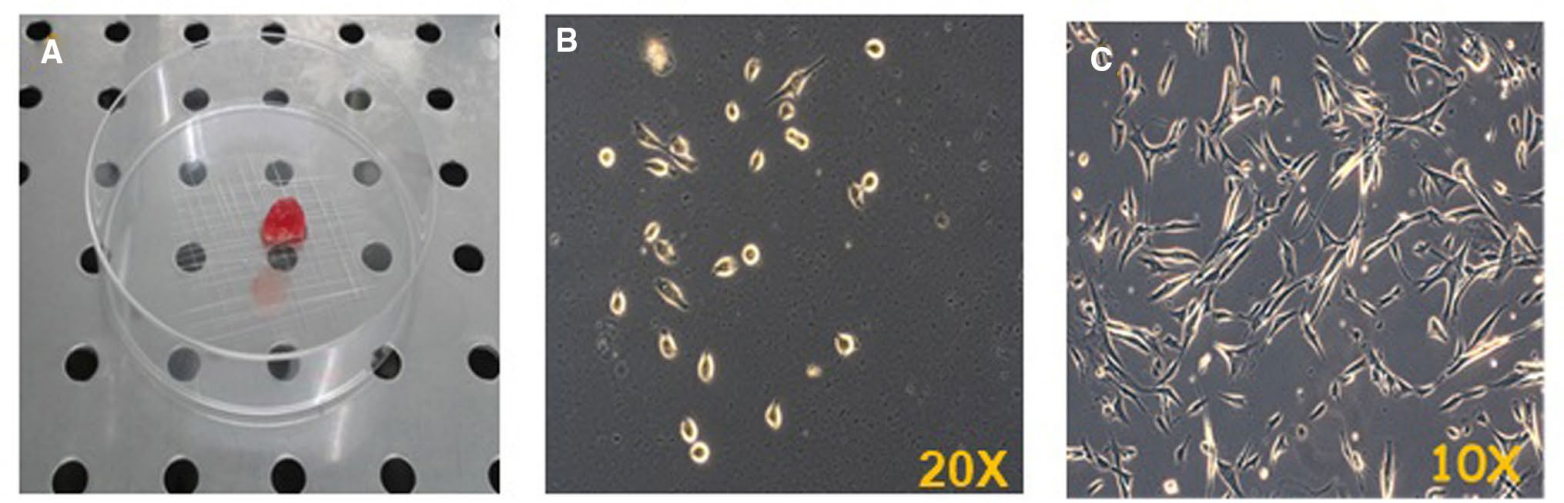
Photo of human skeletal muscle biopsy (**a**); representative observations of primary culture of hSkMCs in phase contrast microscopy at passage 0 after 1 day in the dish; objective ×20 (**b**) and after 3 days in the dish; objective ×10s (**c**)

The cell populations derived from human skeletal muscle biopsies (Fig. 1a), obtained by surgical resection, were amplified in Matrigel® coated plates in order to increase cell adherence and maintain cell phenotype. In fact, the isolated cells, when plated for the first time in a dish (passage 0), attach showing a rounded shape that persists for 2–3 days, with a slow proliferation rate (Fig. 1b). After that period, cells become flatter and show an elongated morphology with 2–4 cytoplasmic extensions (Fig. 1c).

Expanded cells were subsequently characterized by flow cytometry in order to verify their phenotype, analyzing the cluster of differentiation (CD) marker surface proteins (CD44, CD90, CD105, CD56, CD34, CD45) and one of the most reliable markers of the satellite cells (PAX-7).

**Fig. 2:**
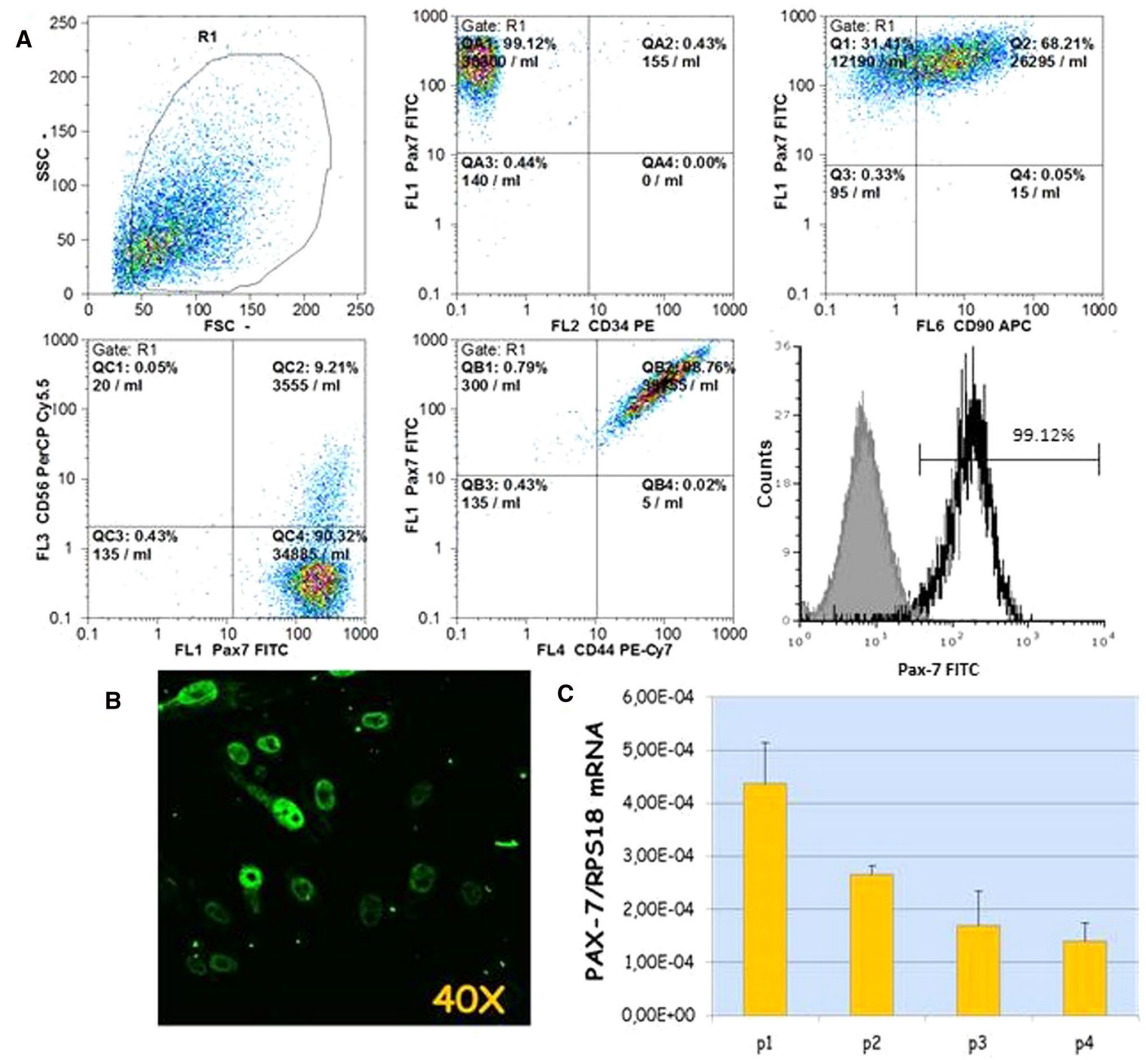
Scattergrams of phenotype characterization of hSkMCs by flow cytometry analysis. The overlay plot shows the percentage of hSkMCs expressing the nuclear marker PAX-7 (open histogram); grey histogram: autofluorescence of unstained cells (**a**); Observation in LSCM of the nuclear transcription factor of satellite cells PAX-7, objective ×40 (**b**); Real-Time qPCR of the PAX-7 gene during passages (from 1 to 4) of hSkMCs. Data are normalized for the housekeeping gene RPS18 (**c**)

The phenotype analysis revealed that isolated hSkMCs expressed the surface markers CD44, CD90, CD105, commonly used to identify mesenchymal stem cells, with a very high percentage of positiveness. In contrast, the hematopoietic lineage marker CD45 was negative. Regarding PAX-7, analysis showed the presence of the nuclear transcription factor in 99.12% of total cells (Fig. 2a). On the contrary, CD34 and CD56, commonly used to identify satellite cells derived from mouse skeletal muscle, turned out to be non-specific for satellite cells derived from human skeletal muscle tissues; in fact, their presence on the cell surface is close to 0% and 9%, respectively (Fig. 2).

Immunofluorescent staining of PAX-7, observed in LSCM, allowed the expression of the nuclear marker to be assessed. As expected from cytometry, it is clearly shown that the PAX-7 protein is present in the nuclei in all the expanded hSkMCs, confirming the isolation of human satellite cells (Fig. 2b).

Moreover, the gene expression of PAX-7 was analyzed over time, to verify its presence at cellular passages used for the experiments. As shown by Real-Time qPCR, we confirmed PAX-7 presence, but, as the passages increase, the gene expression of PAX-7 decreases, reducing the differentiating potential of cells (Fig. 2c). For that reason, we have limited the use of hSkMCs to passage 4.

### Multipotentiality of hSkMCs

The multipotent evaluation of hSkMCs was assessed by the induction toward the adipogenic, osteogenic and myogenic phenotypes, using appropriate media described in “Materials and Methods” section.

**Fig. 3:**
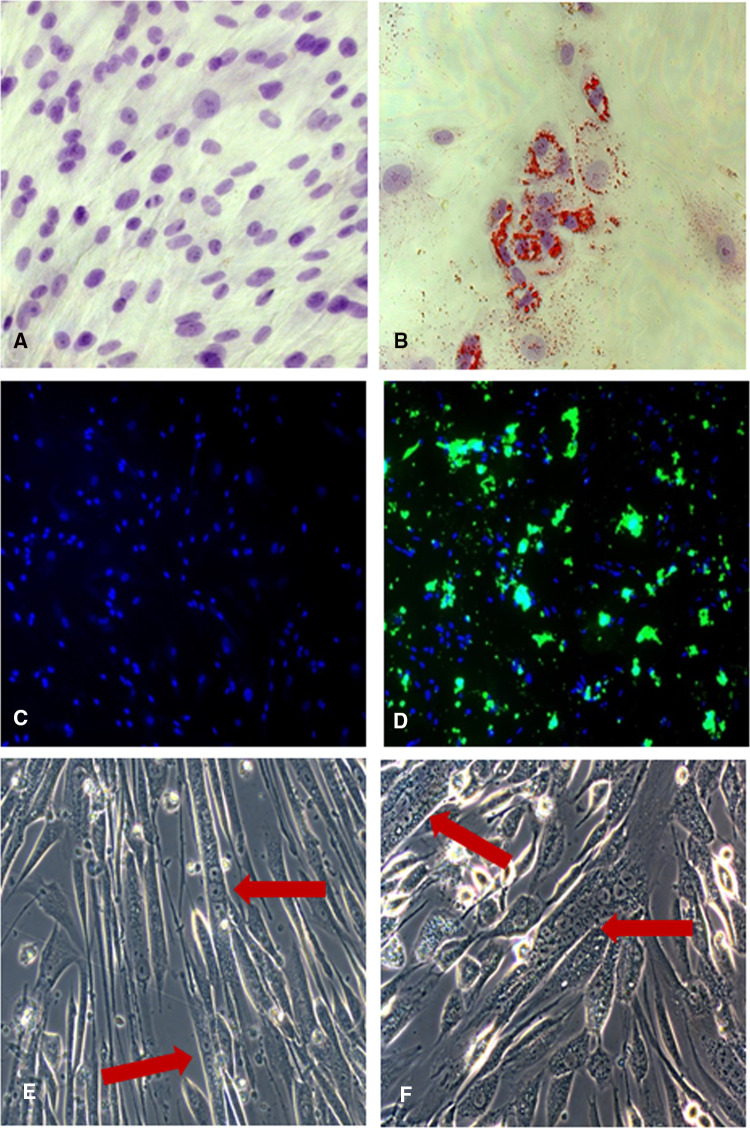
Adipogenic phenotype evaluation of the hSkMCs. Representative images of the adipogenic phenotype evaluation of skeletal muscle-derived cells at time 0 (**a**) and after 7 days of induction (**b**). The intracellular lipid droplets are stained in red by Oil Red O, and nuclei counterstained with Mayer’s acid hemalum in blue. Images acquired in brightfield microscopy. Objective ×20. Osteogenic phenotype evaluation of the hSkMCs. Representative images of calcium staining at time 0 (**c**) and after 15 days of osteogenic induction (**d**). The mineralized calcium deposits are in fluorescent green and nuclei counterstained with propidium iodide in conventional blue color. Images acquired in epifluorescence microscopy. Objective ×10. Representative images in phase contrast microscopy of the multinucleated cells after 9 days of myogenic induction (**e**, **f**). The arrows show the formed myotubes. Objectives ×20

Adipogenic differentiation was performed culturing the hSkMCs in AM for 7 days, and confirmed, using Oil Red O staining, by the multiple intracellular lipid-filled droplets accumulation and microscopic observations in brightfield. In contrast, control cells grown in GM for the same time did not show any formation of lipid droplets (Fig. 3a, b).

Osteogenic induction of hSkMCs was assessed with OM up to 15 days and observed monitoring the production of the mineralized calcium deposits, thanks to the fluorophore calcein added to the medium. Epifluorescence microscopic observations have shown calcein uptake in the calcium nodules after 15 days of osteogenic induction; in contrast, the control cells grown in GM for the same time did not show any deposition of calcium deposits (Fig. 3c, d).

Afterwards, the hSkMCs were differentiated toward the myogenic phenotype using DM for 9 days. During this period, cells started to approach one another, fusing with one another. Observations in phase contrast microscopy have revealed the presence of multinucleated elongated cells (containing from 3 to more than 8 nuclei) referable to myotubes (Fig. 3e, f).

**Fig. 4:**
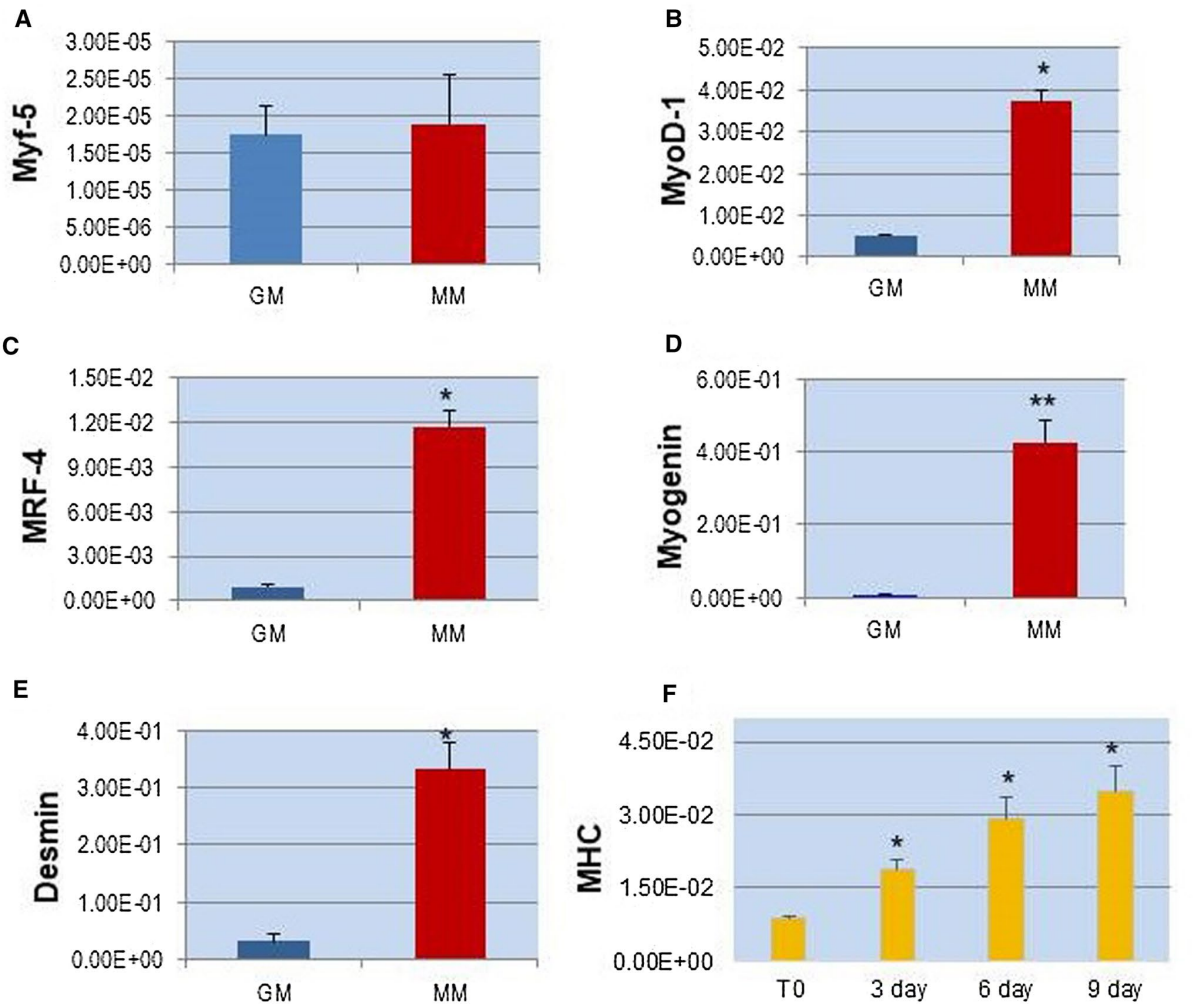
Real-Time qPCR analysis of the main genes during in vitro myogenesis of hSkMCs, in proliferation (red), and after 9 days of myogenic induction in DM (blue). Values are the mean ± SD of 3 independent experiments and they are expressed as the number of mRNA molecules of the genes normalized to the housekeeping RPS18 mRNA. *p < 0.001, **p < 0.005 versus control group in GM

In order to confirm the myogenic induction of hSkMCs, we have performed, using Real-Time qPCR, the analysis of the MRFs (MyoD-1, Myf-5, MRF-4, Myogenin), the main myogenic differentiation genes, Desmin and MHC, after cultivating cells in MM up to 9 days. The results have shown a significant increase in the expressions of these genes during myogenesis, except for Myf-5, with respect to the control in GM (Time 0), indicating that differentiation occurred (Fig. 6). Moreover, differentiation has been supported by the presence of significant increases in the gene expression of Desmin and MHC, which represent essential proteins for proper muscular structure and function (Fig. 4).

**Fig. 5:**
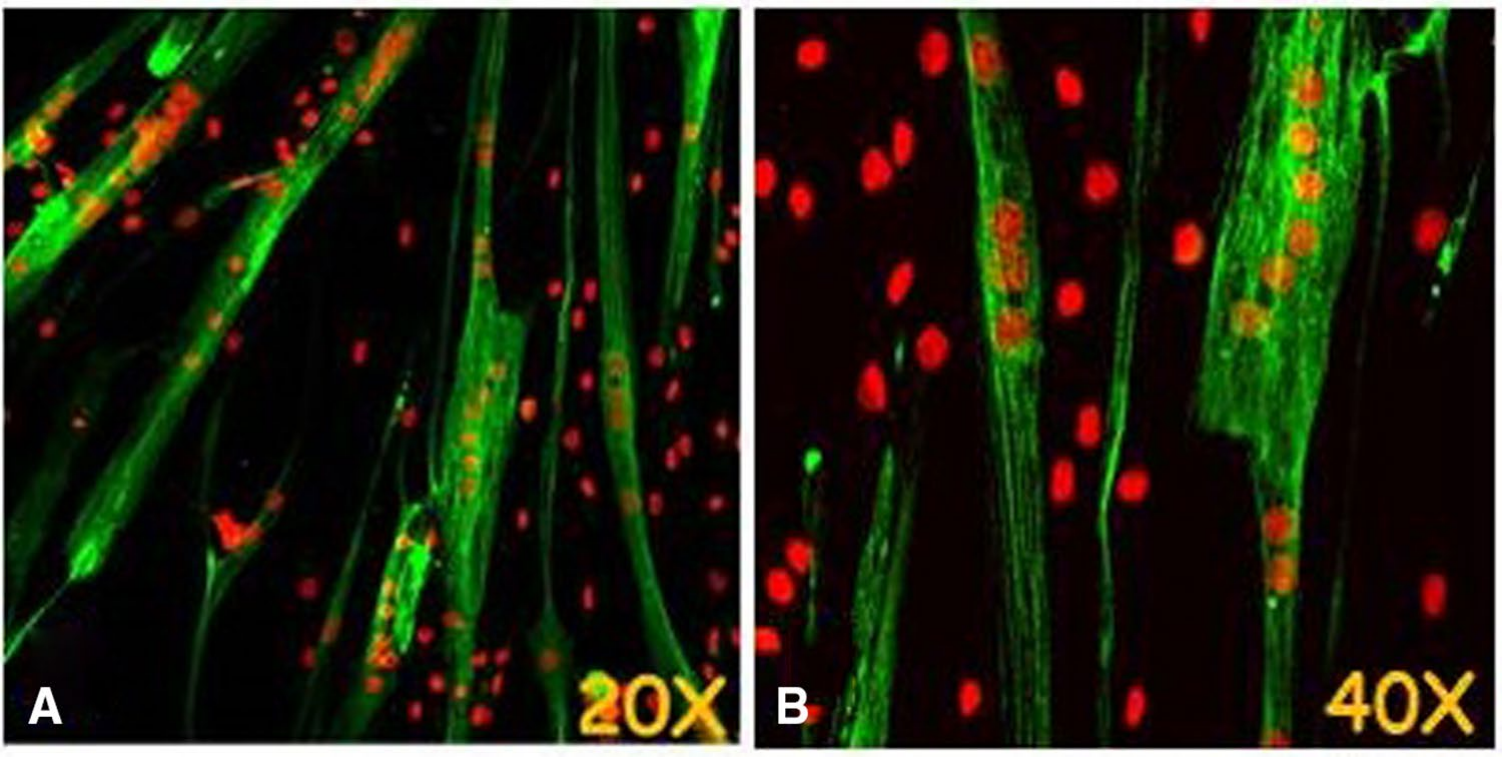
Microscopic observations in LSCM of MHC in hSkMCs after 9 days of myogenic induction (Alexa Fluor 488, conventional green color). Nuclei counterstained with propidium iodide in conventional red color. Objective ×20 (**a**) and ×40 (**b**)

In particular, MHC was assessed at different time points (T0, 3, 6, 9 days) in order to follow the effective myogenesis over time (Fig. 4f). Since MHC is one of the most important proteins in skeletal muscle, and it is essential for contraction and muscle movement, it was analyzed by immunofluorescence staining. The microscopic observation of hSkMCs after 9 days in MM has shown the presence of MHC, demonstrating the suitability of our in vitro myogenesis model (Fig. 5).

### Gene Expression Analysis of Hormone Receptors in hSkMCs During In Vitro Myogenesis

The hSkMCs were differentiated using the MM and, after 9 days of induction, we analyzed the gene expression of the hormone receptors, in order to characterize the maturation and endocrine properties of the cellular model during myogenesis. In particular, we analyzed the following genes by Real-Time qPCR: VDR, TRα, TRβ, GCR, PTH-1R, IFG-1R, LRP-5, LRP-6 and Irisin.

**Fig. 6:**
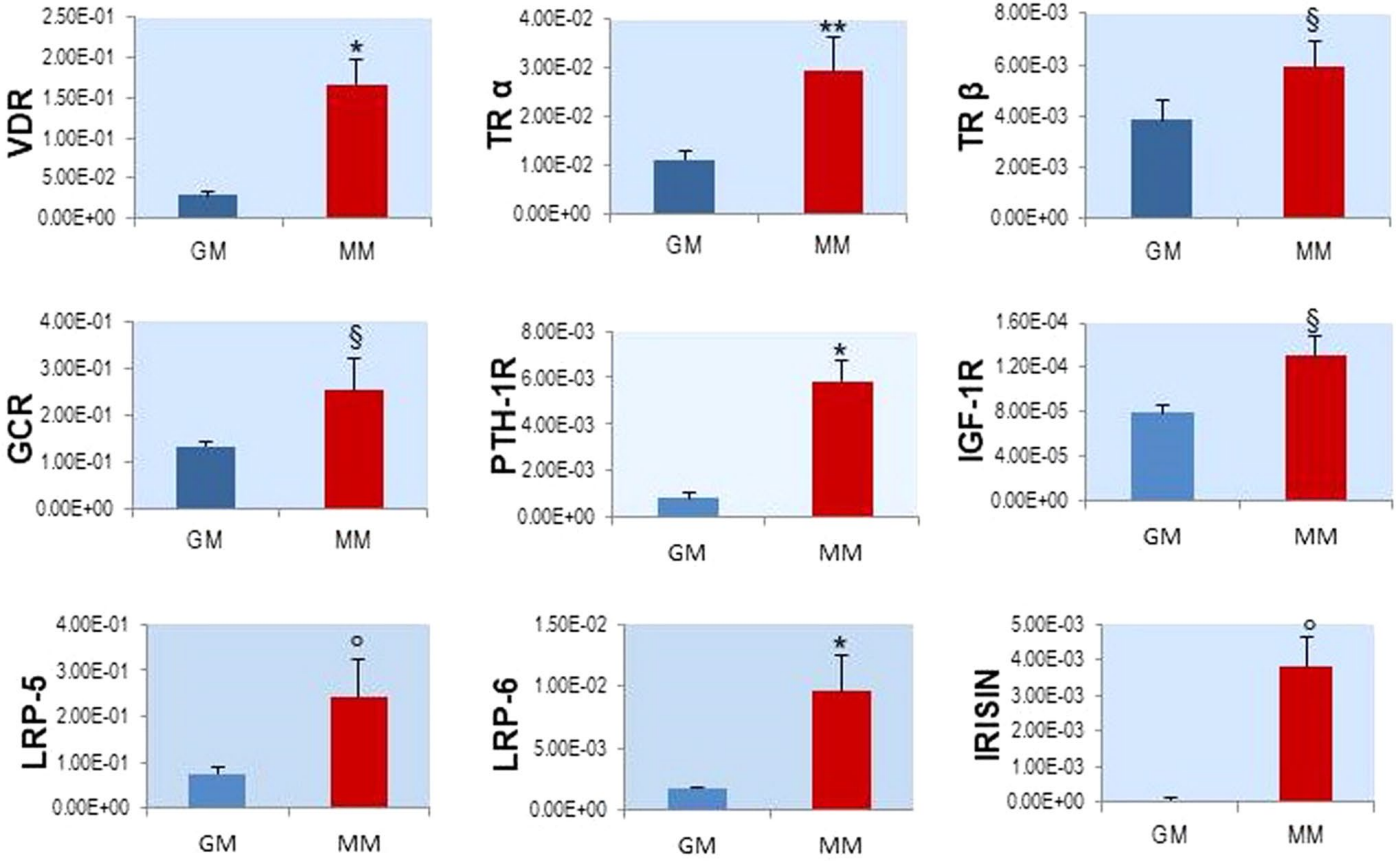
Real-Time qPCR of the hormone receptor genes during in vitro myogenesis of hSkMCs, in proliferation (red) and after 9 days of myogenic induction in DM (blue). Values are the mean ± SD of 3 independent experiments and they are expressed as the number of mRNA molecules of the genes normalized to RPS18 mRNA. *p < 0.001, **p < 0.005, °*p* < 0.01, ^§^*p* < 0.05 versus control group in GM

The results of the hormone receptors analyzed have shown significant increases in gene expressions during cell differentiation with respect to the control group in growth medium, demonstrating the formation of the skeletal muscle as an endocrine apparatus during myogenesis (Fig. 6). Subsequently, the expression of Irisin, a hormone secreted by skeletal muscle, specifically suggests the development and maturation of new myofibers, since it represents a myokine secreted by mature endocrine tissue.

## Discussion

The last decade has been an exciting period for the study of the biology of skeletal muscle stem cells and tissue regeneration and the development of novel human in vitro cell models can contribute to the identification of new mechanisms that control myogenesis [14–20]. These human cell cultures appear more suitable for predictive screening strategies when compared to rodent cell lines, such as C2C12 or rat L6 myoblasts [21, 22].

In this study, we have isolated and characterized skeletal muscle-derived cells from human biopsies to be used for the in vitro study of myogenesis. The developed cellular model was enriched in satellite cells, as confirmed by analyzing the presence of PAX-7 in more than 99% of the hSkMCs and the disappearance of the PAX-7 gene with each passage in culture [23]. For this reason, we decided to use cells not over the fourth passage in vitro.

Although CD34 antigen is a marker of satellite cells in murine models, our results indicate that, in human SCs, CD34 expression is not the hallmark of SCs, as it is present in only 0.5% of hSkMCs [24]. The same is true for CD56 antigen, which is present in only 9% of the isolated hSkMCs, a result that may be controversial considering other data in the literature, since it also marks natural killer lymphocytes [25, 26]. Flow cytometry analysis in hSkMCs has permitted the verification of the presence of the principal markers to be expressed on the surface of mesenchymal stem cells, specifically CD44, CD105 and CD90, and the negativity of the hematopoietic CD45 antigen, confirming the mesenchymal stemness of our cells, as largely recognized in the literature. The multipotentiality of the isolated hSkMCs was confirmed by demonstrating their own capacity to differentiate into the adipogenic, osteogenic and myogenic phenotypes, as assessed by cytochemical staining performed on cells, properly induced with specific differentiating media, as reported in the literature [27].

Using appropriate differentiation medium with confluent cells at 70–80% density in the plate, we were able to induce the alignment of the activated cells (myoblasts), the subsequent fusion with each other and, finally, the differentiation into multinucleated myofibers. Gene expression analyses have shown significant increases of MRFs, MyoD-1, MRF-4, and Myogenin, 9 days after myogenic induction which, together with Desmin and MHC gene expression augmentation during the entire study period, have confirmed myoblast determination and muscle differentiation, confirming the suitability of our in vitro myogenesis model. The only non-significance increase in gene expression was detected for the Myf-5 gene, in agreement with data reported in the literature. The reason may the fact that this transcription factor is the main controller of the activation of SCs toward myogenic differentiation and is expressed in a majority of quiescent SCs in adult muscle [3]. It is also reported that Myf-5, in the absence of MyoD-1, MRF-4 and Myogenin during development, is unable to drive myogenic differentiation, so Myf-5 may contribute to controlling "stemness" in the niche [4] [28].

Once validated the in vitro model, we embarked in the project of characterization of the endocrine machinery in the different stages of myocytic differentiation. This analysis was never performed before in in vitro models. The analysis was focused on selected receptor genes known to mediate a specific hormonal action on skeletal muscle tissue or suggested to mediate an endocrine action on skeletal muscles: VDR, TRα, TRβ, IGF-1R, PTH-1R, GCR, LRP-5 and LRP-6.

In the literature, it has been reported that vitamin D deficiency is a condition associated with skeletal muscle weakness and small muscle fiber size [29]. In animal models, the skeletal muscle dysfunction observed in vitamin D deficiency is reversed by vitamin D repletion, whereas vitamin D supplementation in humans has been found to increase skeletal muscle strength [30]. Many reports suggest that the VDR is expressed in skeletal muscle [31]. VDR deletion in mice results in alterations in muscle function and strength [32], and its association with interleukin-6 may play a role in intramuscular inflammation [33].

The thyroid hormone plays an essential role in myogenesis; it acts as a pleiotropic factor during development and regulates genes involved in growth and differentiation [34, 35]. In particular, data on C2C12 cells and primary myoblasts from mice have suggested the essential role of TRα in the optimal fusion and regeneration of myofibers after muscle injury and to maintain the SC niche during aging [36, 37]. Moreover, it has been reported that TRα is the dominant isoform thyroid receptor in C2C12 and murine primary myoblasts. This is in agreement with our results in which mRNA expression is higher for TRα with respect to TRβ.

Insulin-like Growth Factor-1 (IGF-1), the mediator of growth hormone function, strongly promotes the proliferation and differentiation of skeletal myoblasts. The anabolic effects of IGF-1 are mediated through specific binding with IGF-1R to promote the activation of the PI3K/art/mTOR signaling pathway, which is associated with protein synthesis and muscle hypertrophy [38, 39]. Moreover, IGF-1R is required for normal muscle growth, and its loss on mouse muscle leads to increased basal glucose uptake due to increases in levels of Glu1 and Glu4 transporters, chronic activation of Akt and AMPK signaling, and a loss of TBC1D1, data confirmed also in L6 myotubes [40].

Regarding parathyroid hormone (PTH) and its receptor (PTH-1R), very few data in the literature are reported about their effects on skeletal muscle cells. PTH has been shown to enhance the differentiation of mesoderm to various cell types, including osteoblasts and smooth muscle cells [41, 42]. Since skeletal muscle cells are derived from the mesoderm, it is conceivable that PTH may also influence the differentiation of these cells. It has been reported that PTH and the expression of PTH-1R accelerate the differentiation of SCs to myotubes in a mouse model [43].

Skeletal muscle is a notable target for glucocorticoids (GCs) in health and disease. GCs convey their signals mainly through an intracellular glucocorticoid receptor (GCR). Chronically increased levels of endogenous or exogenous GCs can lead to proteolysis, muscle wasting, myopathy, and induce insulin resistance with severe perturbation in systemic energy metabolism, while short exposures to high GC concentrations have been involved in the development of crucial illness myopathy [44, 45]. Despite its importance, data on GC signaling during human skeletal muscle regeneration and how GCR primary target genes confer metabolic function of GCs remain incomplete [46]. In the literature, it has been reported that GCR is involved in a positive regulation of muscle regulatory gene Myf-5 in the mouse myogenic cell line C2 [47].

LRP-5 and LRP-6 are highly homologous proteins with key functions in canonical Wnt signaling. Alteration in genes encoding these receptors or their interacting proteins is linked to human diseases and, for that reason, they have been a major focus of drug development efforts to treat several human conditions, including osteoporosis, cancer and metabolic disease [48]. Sclerostin is a circulating osteocyte-derived glycoprotein produced by the osteocytes that in a paracrine fashion negatively regulates Wnt signaling after binding the LRP5/LRP6 co-receptors in osteoblastic cells and its pharmacologic inhibition produces bone anabolic effects [49]. Conversely, endocrine effects of sclerostin on muscle morphology remain unknown, and very little data are reported in the literature [50].

In the present study, the genes encoding the receptors for the above outlined hormones were detected in a limited number of collected hSkMCs. Moreover, all the assayed receptor genes significantly increase during in vitro myogenesis of hSkMCs, supporting their role in the maturation of the human skeletal muscle. The fact that the two sclerostin receptors LRP-5 and LRP-6 are expressed in hSkMCs is opens for this important osteocytic protein a function as a hormone in the reciprocal interaction between bone and skeletal muscle. Interestingly, also the expression of the gene encoding Irisin, an important hormone produces by mature skeletal muscle tissue that affects cortical bone [51] increases during the process of in vitro differentiation of the hSkMCs.

## Conclusions

In conclusion, our results have demonstrated the utility of skeletal muscle satellite cells, isolated from human biopsies, as an in vitro cell model to study the myogenesis process and the characterization of the skeletal muscle as an endocrine apparatus and a target organ for several hormones. In fact, all the assayed hormone receptor genes may represent feasible targets during skeletal muscle regeneration to be modulated throughout the skeletal muscle differentiation pathway. Similarly, muscle function should be evaluated in patients suffering of various endocrinopathies, a problem not routinely carried out by the endocrinologists. These findings could stimulate further research for a better understanding of disorders associated with impaired adult myogenesis, to help identify novel therapeutic interventions for these conditions. Future developments of this research will include the enlargement of the culture collection and the characterization of the novel evidenced targets in human skeletal myogenesis.

## Abbreviations

VDR: Vitamin D receptor
TRα: Thyroid receptor α
TRβ: Thyroid receptor β
GCR: Glucocorticoid receptor
PTH-1R: Parathyroid hormone receptor-1
IFG-1R: Insulin-like growth factor 1 receptor
LRP-5: Low-density lipoprotein receptor-related protein 5
LRP-6: Low-density lipoprotein receptor-related protein 6
PAX-7: Paired box protein 7
MRFs: Myogenic regulatory factors
MHC: Myosin heavy chain
hSkMC: Human skeletal muscle-derived cells
SCs: Satellite cells
